# Thermovision as a Tool for Athletes to Verify the Symmetry of Work of Individual Muscle Segments

**DOI:** 10.3390/ijerph19148490

**Published:** 2022-07-12

**Authors:** Agnieszka Szurko, Teresa Kasprzyk-Kucewicz, Armand Cholewka, Maksymilian Kazior, Karolina Sieroń, Agata Stanek, Tadeusz Morawiec

**Affiliations:** 1 Faculty of Science and Technology, University of Silesia, 75 Pułku Piechoty 1A, 41-500 Chorzów, Poland; teresa.kasprzyk-kucewicz@us.edu.pl (T.K.-K.); armand.cholewka@us.edu.pl (A.C.); kazior.max@gmail.com (M.K.); 2 Department of Internal Diseases, Oncology, with Department of Gastroenterology, Cardiology, and Angiology, Hospital MSWiA in Katowice, 40-752 Katowice, Poland; ksieron@sum.edu.pl; 3 Department and Clinic of Internal Medicine, Angiology and Physical Medicine, Faculty of Medical Sciences in Zabrze, Medical University of Silesia, Batorego 15, 41-902 Bytom, Poland; astanek@tlen.pl; 4 Division of Medicine and Dentistry, Department of Oral Surgery, Medical University of Silesia, Pl. Akademicki 17, 41-902 Bytom, Poland; tmorawiec@sum.edu.pl

**Keywords:** thermovision in sport, body efficiency, symmetry of muscle work, thermal characteristics of muscles, exercises on a rowing ergometer

## Abstract

In the presented research, we characterised the temperature profiles and the degree of preparation for exercise of individual muscle groups of athletes We hypothesise that by means of thermal imaging studies, the effectiveness of the warm-up can be monitored to determine whether the effort of individual muscles is equal and symmetrical, which can help to avoid a potential injury. In the study, thermographic imaging was performed on a group of athletes exercising on a rowing ergometer involving almost 80% of the muscle parts of the human body for intense and symmetrical exercise. Thermovision studies have confirmed, based on the increased temperature of the muscle areas, that the rowing ergometer involves many muscle groups in training. Moreover, based on the shape of the temperature function obtained from individual body regions of interest, it was shown that conventional exercise on a rowing ergometer causes almost symmetrical work of the right and left sides of the body. Obtained temperature changes in most of the studied muscle areas showed minimum temperature reached the beginning of training—mostly phases 1 and 2. During the subsequent phases, the temperature increase was monitored, stopping at resting temperature. Significantly, temperature variations did not exceed 0.5 °C in all post-training phases. Statistical analyses did not show any significant differences in the symmetry of right and left muscle areas corresponding to the muscle location temperature. Thermal imaging may be an innovative wholly non-invasive and safe method, because checking induces adaptation processes, which may become indicators of an athlete’s efficiency. The imaging can be continuously repeated, and automatic comparison of average temperature or temperature difference may provide some clues that protect athletes from overtraining or serious injuries.

## 1. Introduction

The distribution of body temperature and effector mechanisms have long been recognized. Temperature sensors are widely distributed throughout the body, which has a progressively ascending temperature control system [1]. The skin temperature in each body area is determined by the thermal balance between muscle activity, vasodilation, the rate of sweat evaporation and the environment [2,3,4,5]. Thus, core and mean skin temperature are often considered the regulatory parameters [6]. Infrared thermography is an excellent non-contact tool for assessing skin temperature, with the ability to map the distribution of temperature throughout the body [6]. Thus, thermal infrared imaging provides the basis for evaluating local and systemic cutaneous blood flow adaptation as a function of a specific type, intensity and duration of exercise and helps to determine the ideal conditions (in terms of environment and apparel) in which physical activities should be performed to favour thermal regulatory processes [7].

One of the methods used in sports medicine to monitor exercise processes and diagnose trauma is thermography [8,9,10]. Thermography also plays a vital role in post-workout recovery due to its ability to show body map temperature changes. The regenerative mechanisms regulated by thermal factors and the return to the body’s homeostatic resting temperature are primarily conditioned by the blood system’s response to physical activity. In turn, changes in the activity of the blood system in the muscles and, of course, on the surface of the body lead to changes in the thermal map of the body [11].

Creating effective athlete training requires registration of the so-called ‘Physiological measures of effort’. For this reason, athletes undergo endurance tests that assess the body’s physical condition. When testing the body’s efficiency, it is necessary to simultaneously measure a number of parameters that determine the body’s efficiency (e.g., lactic acid level, respiratory parameters), ranging from easily measurable parameters, such as heart rate, to those whose measurement is more complicated [12].

Thermographic analysis of the athlete’s body surface can be irreplaceable in the overall assessment of the body’s efficiency. Thermovision can easily analyse the changes taking place in the body and thus can contribute to the appropriate selection of exercises for the athlete. Thermovision can also be helpful in preventing the phenomenon of overtraining [13,14,15,16,17]. Thermovision has been used both to assess the level of physical fitness [18,19] and the effectiveness of the warm-up in many sports disciplines [20,21,22]. The effectiveness of thermal imaging in fitness level assessment was shown in a study where the mean body surface temperature was compared for cycling volunteers before and after one year of professional training. Initial thermal images did not indicate a relationship between the thermal map and the level of physical fitness for the studied group of volunteers. However, a significant difference in the values of the average temperature of the athlete’s body was found comparing thermal images obtained before the start of the annual training period and after a completed training period. Such results proved that the differences in the body surface temperature of two groups of athletes undertaking effort might be related to the physiological state of their organisms obtained as a result of training. The body temperature differences lead to the conclusion that these sportsmen differ in efficiency [19].

Intensive training, especially in endurance athletes, is associated with a high load on the muscles and their vibration, which in turn leads to micro-damages (i.e., damage to structural proteins in muscle fibres or connective tissue) and the formation of local inflammations that turn into pain [23]. Muscle damage during exercise develops when the limit of adaptation of a given muscle to the mechanical training loads imposed on it is exceeded. Next, disturbances in calcium homeostasis and induction of inflammatory mediators occur, intensifying muscle damage within hours or days after exercise (DOMS—Delayed Onset Muscle Soreness). These changes can be seen at the biochemical level in the peripheral blood by indicators of muscle damage such as creatine kinase (CK) and inflammatory biomarkers (C-reactive protein (CRP) and interleukin-6 (IL-6)) [23,24,25].

Therefore, it is imperative that sports trainers and scientists optimise the recovery period of the athlete’s muscles. Sufficiently efficient regeneration will prevent muscle damage and alleviate DOMS, inflammation and fatigue, thus reducing the risk of injury or maladjustment to the training load. Moreover, more research using thermal imaging is still needed to provide coaches, and physicians in sports injuries and regenerative medicine, with complete qualitative and quantitative information about the health and condition of athletes. Thus, modern technologies such as thermal imaging can significantly improve the monitoring of athletes’ preparation, indirectly contributing to increasing the level of competition.

Our paper presents thermal imaging studies on a group of athletes exercising on a rowing ergometer. The objective was to create a body surface thermal map of a trained person (professional athlete). Since it has been known for a long time that increased muscle temperature may also be one of the mechanisms in the cascade of events leading to the formation of DOMS [26], we want to characterise the temperature profile in individual muscle groups in our research. We hypothesised that by employing thermal imaging tests, we could monitor the warm-up effectiveness process and determine that the effort of individual muscles is uniform and symmetrical, which may help avoid a potential injury. Our thermographic examinations will facilitate observation of the work of muscles and their return to a state of rest, which is the basis of post-workout recovery.

## 2. Materials and Methods

### 2.1. Participants

Thermal imaging studies were carried out on a group of 6 volunteers. They were male adults with ages ranging from 24 to 30 years. The research group of professionals was characterized by a high degree of training. This includes people who have completed at least three training units per week. Additionally, these people had systematically maintained this high level of training for at least two years. The characteristics of the research group are presented in Table 1.

The research was carried out on people who declared good psychophysical condition in the initial interview and were healthy on the day of the examination. Before starting the measurements, each of the volunteers carried out a short individual warm-up in order to prevent an injury. After the initial start-up, each study participant began the 30 min acclimatization phase. In this phase, acclimatization to the conditions in the test room took place. The adaptation of volunteers consisted of staying at rest with parts of the body that were included in the measurement protocol exposed, i.e., the lower and upper limbs, back, chest and neck.

### 2.2. Research Room

The research room was prepared according to The Interactive Thermology for Europe (IATE) guidelines. The minimum area of such a room should not be less than 6 m^2^, keeping the dimensions of the walls as 3 × 2 m. However, conducting tests in larger rooms is recommended, which was fulfilled in our case. There were no heat emitters in the test room, as they can largely induce thermolysis processes of the examined person and create an air barrier with increased temperature between the IR camera lens and the imaged object. Similarly, there were no devices inducing air circulation in the room, as they affect the thermogenesis processes of the examined person [27]. Moreover, IATE recommends that air humidity be kept at the level of 45 to 55%, and the measurable air temperature should be between 20 and 22 degrees Celsius [27,28]. In our case, the parameters of ambient temperature and humidity, recorded using a classic electronic thermometer with the option of a hygrometer, were respectively 19.9 °C ± 0.6 °C and 45.1% ± 2.1% (instrument measurement uncertainty: ΔT = 0.1; Δf = 0.1%).

### 2.3. Qualification and Measurement Procedure

Each person participating in the study joined it voluntarily. People were qualified based on a questionnaire, which was an indispensable element of preparation for the thermographic examinations. The questions concerned issues related to the consumption of pharmaceutical products, stimulants and participation in sauna and physiotherapy sessions within the five days preceding thermographic imaging. All persons passed the interview and started the experiment.

Physical activity on the rowing ergometer was carried out according to a specially prepared protocol. The rowing ergometer seems to be a convenient exercise trainer, as it employs almost 80% of the muscle parts of the human body. Notably, during exercise on a rowing ergometer, individual muscle segments along the sagittal plane passing through the long axis of the human body are symmetrically involved in the effort [29,30]. Therefore, the assumption of the training was its high intensity, engaging as many muscles as possible in a short time and, moreover, that the training was similar and repeatable for each participant. A three-minute effort was planned on the rowing ergometer; each time, the resistance element was set at 90% of its capacity. The trainer used is the Concept 2 Model D, which is also the most popular rowing ergometer in scientific research in the field of sports medicine.

Thermographic imaging was carried out according to a predetermined scheme. In the beginning, a measurement was made before performing physical activity, which is the baseline measurement of the body surface temperature (hereinafter referred to as PHASE 0). Then, immediately after taking the infrared images, physical activity was carried out, and immediately after it, another thermal imaging measurement was performed (PHASE 1). Finally, the measurement of the recovery phases was carried out according to the scheme from 10 to 50 min after training, keeping the interval between the imaging series of 10 min (PHASE 2 to 6).

In the research, a Flir System T640 thermal imaging camera with a temperature sensitivity of 0.03 K was used. The measurement system consisted of an infrared camera that was placed on a tripod and a designated place for a volunteer at a distance of 3.0 ± 0.1 m.

The measurements of the volunteer’s body weight as well as the percentage of body fat were carried out using the TANITA UM-018 multifunction electronic scale. The weight measurement uncertainties were specified by the manufacturer as 0.1 kg for body weight measurement and 0.1% for the percentage of adipose tissue in the body volume. Measurement of the height of the test participants and determination of the distance of the measuring system was made using a classic length gauge, the measurement error of which was 0.01 m.

The average temperature of the determined ROI covering the given muscle parts was calculated according to the formula below from all the temperature values of the pixels constituting the designated analysed areas.

(1) $$T_{ŚRx}=\frac{\sum t_{n}}{n}$$

where *t_n_*—temperature value of a given pixel, and *n*—number of pixels constituting the designated area.

The chosen areas of interest (chosen muscle parts) plotted on the recorded measurement thermograms were created on the basis of the Thermotracker software database, specialized in this field (pemaGROUP company). The algorithm is dedicated to archiving 78 surface areas from the whole human body volume collective.

**Figure 1:**
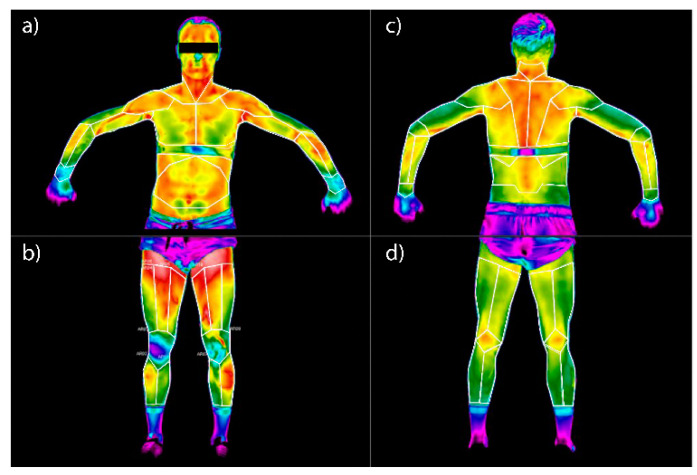
Regions of Interest (ROI) on the surface of the human body used in the research: (**a**) anterior surface of the chest and upper limbs, (**b**) anterior surface of the lower limbs, (**c**) posterior surface of the chest (back) and upper limbs, (**d**) posterior surface of the lower limbs.

For the analysis undertaken in this study, calibration was used to the positions of the imaged volunteers and the surfaces analysed (Figure 1). Significant areas of interest in this work were the skin surfaces of the lower and upper limbs, the chest and abdomen, and the back and neck. The areas on the thermograms were marked manually. However, care was taken to ensure that the individual surfaces did not overlap and that the background was separated by keeping a small margin of a few pixels and not going beyond the registered surface of the volunteer body during the contouring operation.

The outlined anatomical areas of interest are numerically assigned and correspond to the given muscle parts (Figure 1). The correlation is as follows: 1—right wrist elbow flexor, 2—left wrist flexor, 3—right biceps muscle, 4—left biceps muscle, 5—left triceps muscle, 6—left triceps muscle, 7—right pectoral muscle, 8—left major pectoral muscle, 9—right anterior dentate muscle, 10—left dentate anterior muscle, 11—right rectus muscle, 12—right trapezius muscle, 13—left trapezius muscle, 14—gastrocnemius muscle right, 15—left gastrocnemius muscle, 16—right quadriceps muscle, 17—left quadriceps muscle, 18—right deltoid muscle, 19—left deltoid muscle, 20—right latissimus dorsi muscle, 21—left latissimus dorsi muscle.

Recorded images representing the temperature maps of the human body surface were made using a specialized thermal imaging camera, correlated with dedicated ThermaCAM TM Researcher Pro software, version 2.8. Pixel temperature values from Region of Interest (ROI) were then exported to spreadsheets. Using Microsoft Office Excel version 2007, measurement data analysis, statistical analysis and plotting of time courses of changes in temperature parameters obtained from the obtained thermal images were carried out.

The differences between mean temperature as well as normality temperature parameters obtained for each phase in every symmetrical pair of muscles were checked by using statistical analysis. Our explorative data analysis consisted of three main analysis steps. First, the Shapiro-Wilk’s test was used to check for normality, then Levene’s test to check for homogeneity of variances and finally, in case of normal distribution, t-test was performed, and Wilcoxon’s test if not. The significance level was set at α ≤ 0.05 for all statistical tests. All the statistical analyses were conducted with Statistica 12 software.

## 3. Results

Thermal imaging was performed on a group of volunteers, dividing the entire process into several phases. The measurement phases were defined as follows: PHASE 0—measurement at rest before performing an exercise on an ergometer, PHASE 1—imaging immediately after physical activity, PHASE 2—the first interval after 10 min from physical activity, PHASE 3—measurement after 20 min from activity, PHASE 4—measurement 30 min after the end of the exercise, PHASE 5—measurement 40 min after the end of the exercise, and PHASE 6—measurement 50 min after the exercise. PHASE 0 is taken as the body’s base temperature. PHASE 1 measures the direct effect of exercise on the body. STAGES 2 to 6 are called regeneration steps, as the body works through physiological processes to achieve thermal homeostasis and achieve its nominal temperature. Any deviation from the baseline by 0.5 °C is taken within the tolerance as an appropriate indication.

The course of the mean temperature changes for each anatomical surface of interest was subjected to the normalisation function. The base temperature was measured in phase 0. Each successive temperature value in each measurement phase was normalised to the baseline measurement according to the following formula:

ΔT = T_ (PHASE 0) − T_ (PHASE X)(2)

where ΔT—change in the mean temperature value of a given region of interest, T PHASE 0—mean body surface temperature of the region of interest before physical activity (PHASE 0), and T PHASE X—mean temperature of a given region of interest in subsequent measurement steps (PHASE from 1 to 6).

The results of changes in the mean temperature for each measurement phase, along with its uncertainty determined by the standard deviation method, are presented in Table 2.

**Figure 2:**
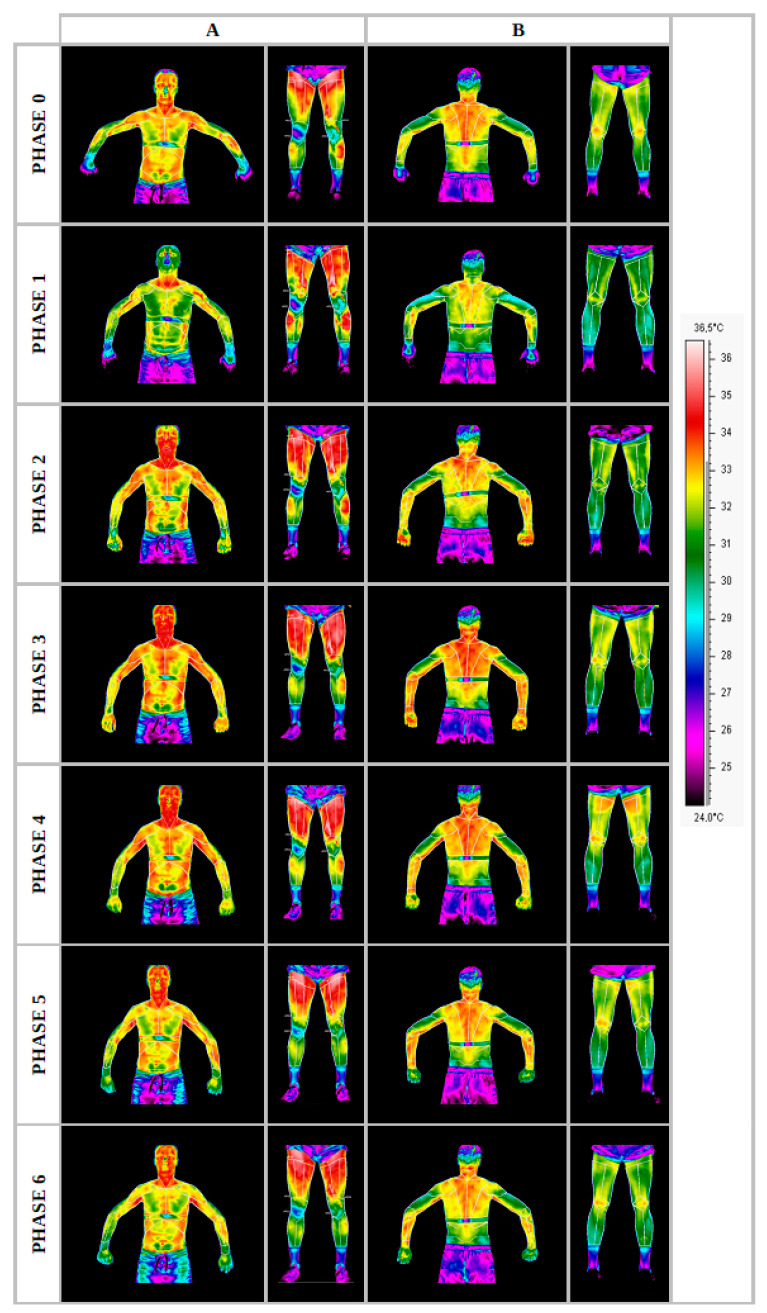
Thermograms representing the temperature maps of the front (**A**) and rear (**B**) body surfaces of a professional group representative, recorded in each measurement phase. Source: own research carried out following the methodology described.

The course of changes in temperature distributions on the body surfaces was presented in the form of thermograms, separating the front surface (Figure 2A) from the back surface (Figure 2B) for one randomly selected representative of the group. The remaining thermograms were similar to the examples (Figure 2). However, the normalized temperature changes obtained for the group of professionals are presented in Table 2.

**Figure 3:**
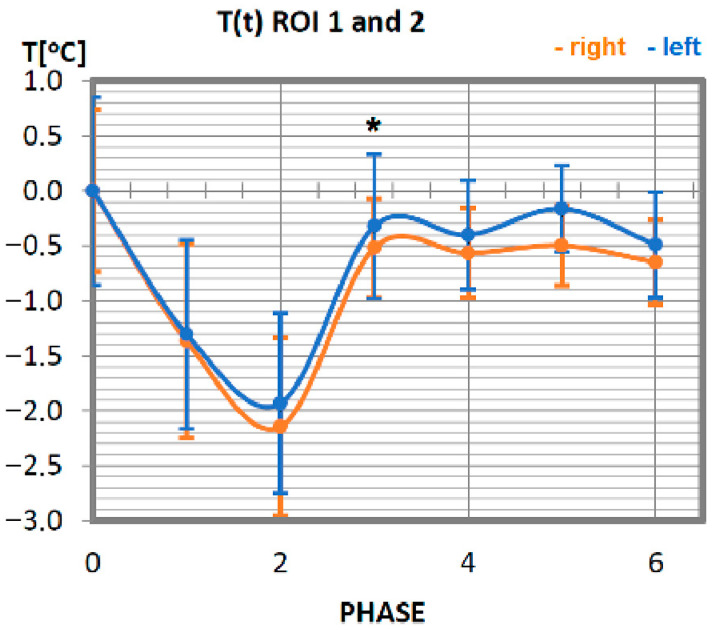
Temperature time (0 to 6 phase of training according to methods) changes of the wrist elbow flexor muscles obtained before and after training using a rowing ergometer for the studied group of athletes. The largest but not statistically significant differences were marked with *, *p* = 0.08.

**Figure 4:**
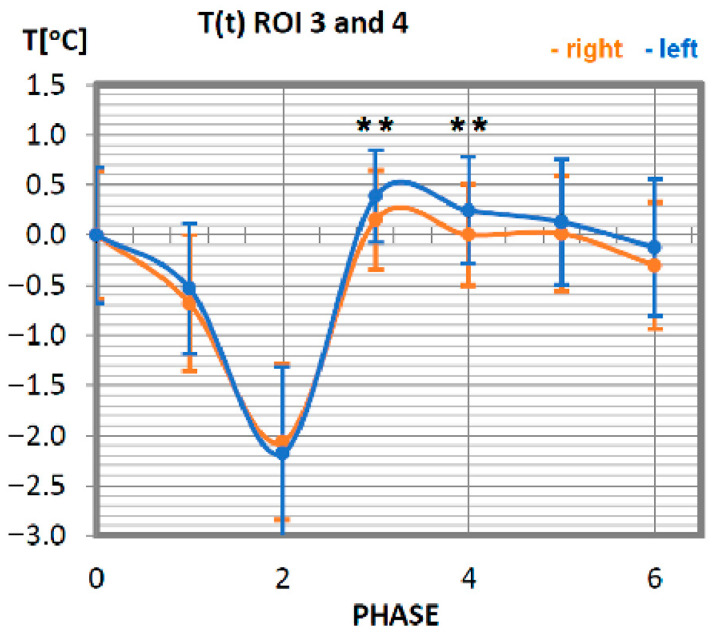
Temperature time (0 to 6 phase of training according to methods) changes of the biceps brachial muscle obtained before and after training using a rowing ergometer for the studied group of athletes. The statistically significant differences were marked with **, *p* = 0.04.

**Figure 5:**
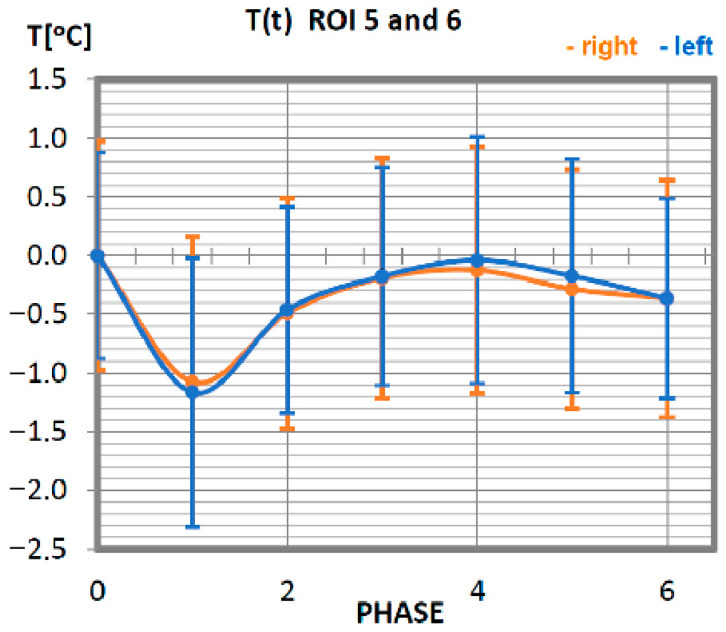
Temperature time (0 to 6 phase of training according to methods) changes of the triceps muscle of the arm obtained before and after training using a rowing ergometer for the studied group of athletes.

**Figure 6:**
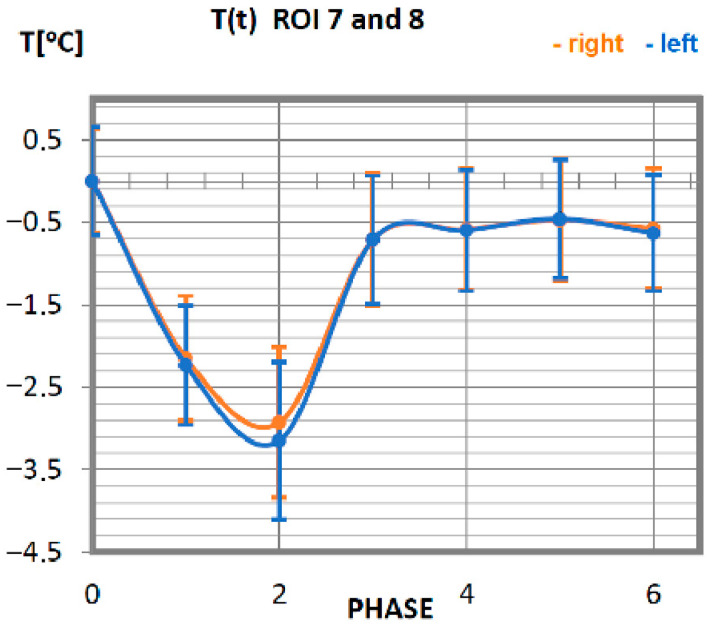
Temperature time (0 to 6 phase of training according to methods) changes of the greater pectoral muscles obtained before and after training using a rowing ergometer for the studied group of athletes.

**Figure 7:**
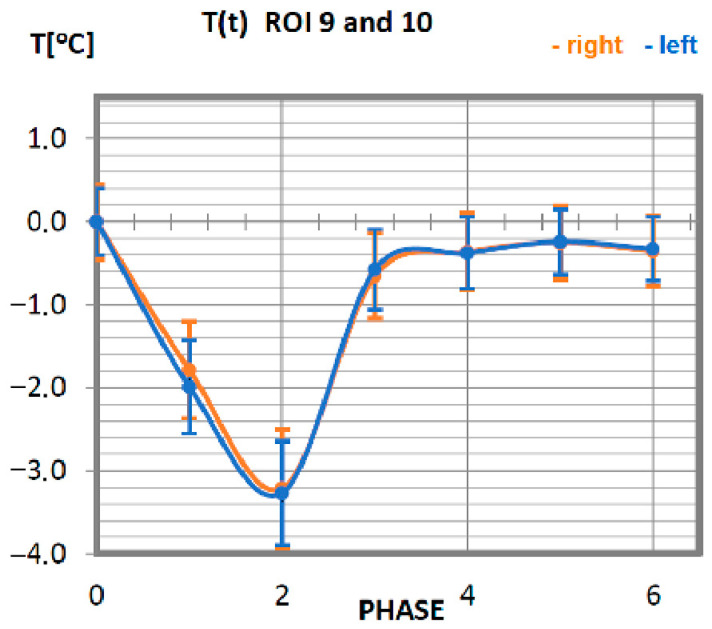
Temperature time (0 to 6 phase of training according to methods) changes of the anterior dentate muscle obtained before and after training using a rowing ergometer for the studied group of athletes.

**Figure 8:**
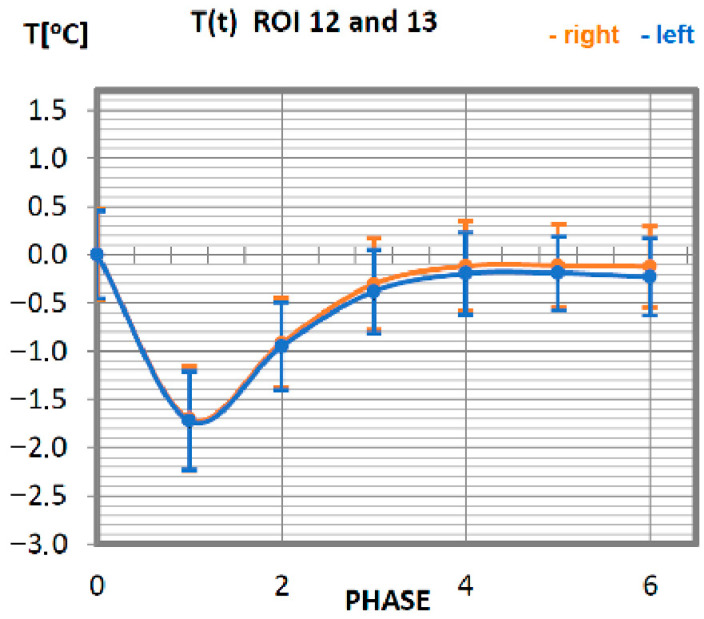
Temperature time (0 to 6 phase of training according to methods) changes of the trapezius muscle obtained before and after training using a rowing ergometer for the studied group of athletes.

**Figure 9:**
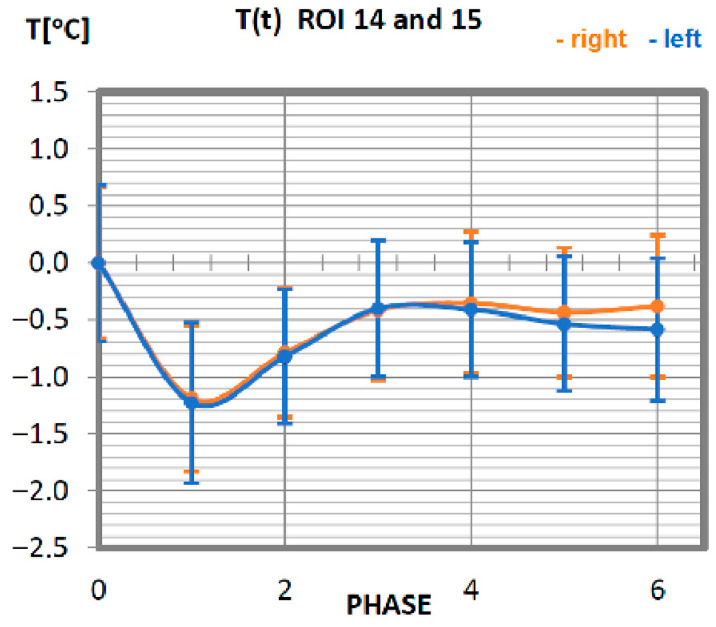
Temperature time (0 to 6 phase of training according to methods) changes of the gastrocnemius muscle obtained before and after training using a rowing ergometer for the studied group of athletes.

**Figure 10:**
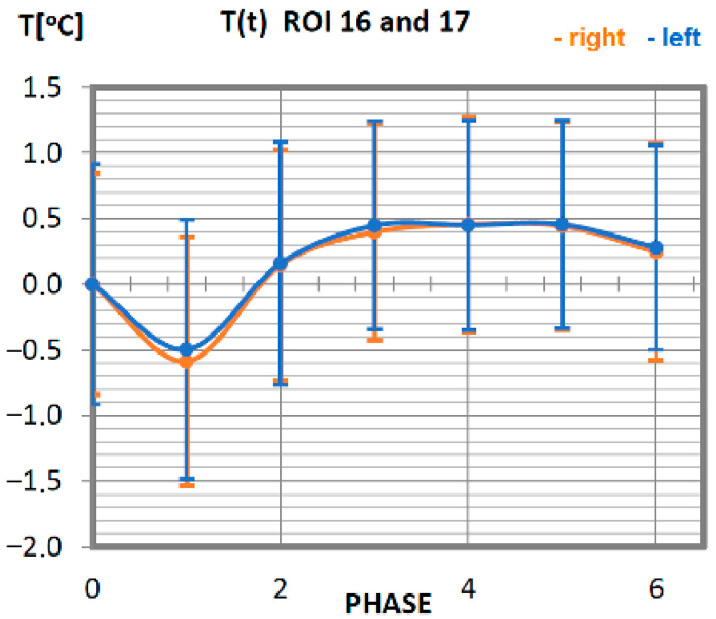
Temperature time (0 to 6 phase of training according to methods) changes of the quadriceps muscle obtained before and after training using a rowing ergometer for the studied group of athletes.

**Figure 11:**
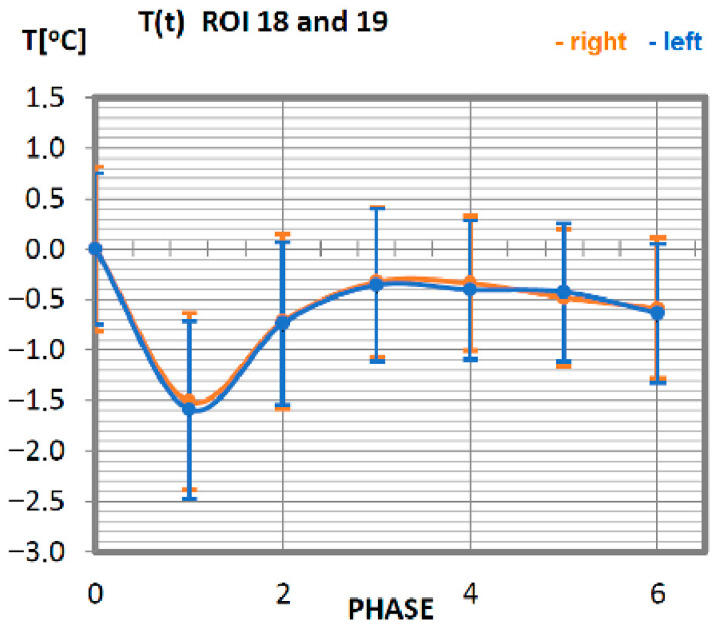
Temperature time (0 to 6 phase of training according to methods) changes of the deltoid muscle obtained before and after training using a rowing ergometer for the studied group of athletes.

**Figure 12:**
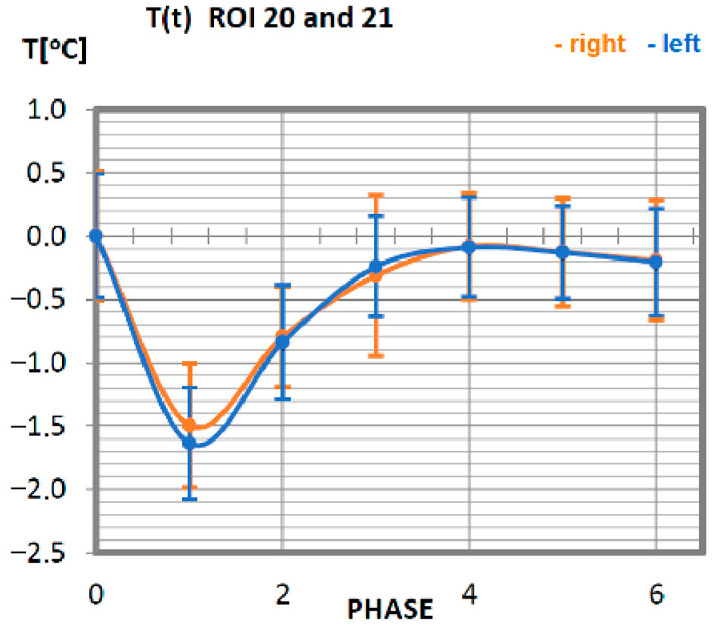
Temperature time (0 to 6 phase of training according to methods) changes of the latissimus dorsi muscle of the thigh obtained before and after training using a rowing ergometer for the studied group of athletes.

Carrying out the obtained results, a detailed analysis of the temperature changes occurring in individual areas of interest was performed, which were assigned topologically to the corresponding muscle parts, symmetrical to the sagittal plane, passing through the long axis of the spine (ROI 1–10, 12–21). These changes are presented in Figure 3, Figure 4, Figure 5, Figure 6, Figure 7, Figure 8, Figure 9, Figure 10, Figure 11 and Figure 12, plotted based on the measurement data collected in Table 2.

Changes in the temperature of the wrist flexor muscle for a group of professionals are presented in Figure 3. Initially, that is immediately after exercise (phase 1) and after a 10-min break (phase 2), a tendency to decrease in temperature was shown as a result of physical activity until it reached its minimum value in phase 2 (10 min after exercise). Thereafter, an increase in temperature is observed, which, from the third phase (20 min after training) to the end of the test period, remains at the level of the value before the physical activity. When analysing the symmetry of muscle involvement, taking into account the orientation sides of the human body surface, in most examples lower temperature values were recorded for the left side. Slightly higher values of temperatures were measured on the right side. Still, due to their slight divergence from the left side, it can be assumed that the wrist flexor muscles were engaged in the effort in the same way (no statistical significance was obtained in every single phase), working symmetrically in relation to the long human axis.

Changes in the temperature of the biceps muscle also show that the temperature symmetry of these muscle parts is maintained. The temperature of the biceps brachial muscle in advanced athletes decreases until phase 2 (10 min after exercise), reaching its minimum at a value 2 °C lower than the initial temperature. This tendency is maintained for the muscle parts located on both sides. Thereafter, at 20 min after exercise (phase 3), there is a sharp rise in temperature, observable until the nominal muscle temperature at rest is reached. The next measurement phases represent the muscle temperature that does not differ from the temperature before exercise.

Characteristics of average temperatures of the triceps muscle in professionals are shown in Figure 5. The minimum temperature was recorded in the measurement taken immediately after the training unit was completed, i.e., in phase 1. The group of professionals was characterised in this phase by a decrease in the average temperature of the measured area, at the level of approx. 1 °C. Thermal homeostasis occurred 10 min after physical activity. The discrepancy in the characteristics of the right triceps brachium from the left triceps brachium is small, which proves the symmetrical work of both groups.

The mean values of the temperature of the pectoral muscles as a function of time are presented in Figure 6. Achieving the minimum value of the temperature in professionals is observed after 10 min of performing an activity on a rowing ergometer. After reaching the lowest reading of the temperature, it increases. The return to the nominal temperature of the body (taking into account the measurement error of approx. 0.5 °C) occurs in advanced athletes in phase 3. The symmetry of the work of the muscles located on the left side to their counterparts on the right side is maintained.

The temperature characteristics of the anterior dentate muscle as a function of time are presented in Figure 7. ROI 9 and 10 temperatures for professionals take a declining course up to 10 min after training (phase 2), reaching its minimum, regardless of the side, for a value lower by approx. 3.5 °C from the nominal body temperature. Thereafter, the temperature rises until the 20th min after training (phase 3), where it stabilises at a value close to the temperature at rest.

The temperature course of the trapezius muscle of the professional group (Figure 8) is quite orderly. The symmetry of changes in relation to the long axis of man is preserved. The minimum temperature value is measured directly after training (phase 1). The trapezius muscle of this research group obtains its base temperature 20 min after exercise, and this temperature does not change any further.

The analysis of the function of changes in the temperature of the gastrocnemius muscle of professionals is presented in Figure 9. The minimum temperature value was obtained in phase 1. The thermolysis processes last up to 20 min after the work on the rowing ergometer. For all stages of post-training temperature measurement in the group of professionals, the indications are lower than the temperature measured before exercise. The symmetry of the work of the muscles is preserved in relation to the long axis of the human being.

The recorded changes in the temperature of the quadriceps in response to training are shown in Figure 10. The minimum temperature occurs for phase 1 (direct measurement after training). In the following phases, the temperature increases to reach the resting temperature. Beginning with phase 2, the temperature of the muscle starts to be positive, staying at the level not exceeding 0.5 °C, which allows the assumption that this is the nominal value. Temperature variations not exceeding 0.5 °C occur for all post-training phases, so it can be assumed that the quadriceps muscle did only a small amount of work in this training in relation to the total power it can generate. The symmetry of the work of the right and left muscles has been preserved due to the same course of functions corresponding to the sides of muscle location.

Temperature changes in response to exercise, performed on a rowing ergometer for the deltoid muscle of professional athletes (Figure 11), are ordered. The temperature corresponding to the surface area occupied by the deltoid muscle reaches its minimum immediately after the training unit (phase 1), and the recovery time of the muscle at rest takes 20 min. The deltoid muscles work with the same intensity because the courses of thermal characteristics of the muscle parts located on the right-side overlap with the techniques of the characteristics of the left side.

In the last analysis, temperature changes in the skin surface within the latissimus dorsi muscle were determined (Figure 12). Symmetry is maintained for the muscles on the right side in relation to the left side of the body. The minimum temperature value occurred for phase 1. Return to the nominal temperature of the organism takes place for a group of professionals in phase 3.

## 4. Discussion

Exercising on a rowing ergometer requires special attention to technique. Although the movement seems easy to repeat, it consists of several phases, the sequence of which is crucial and determines the effectiveness of the exercise. Some muscles, such as the muscles of the legs, are engaged earlier than others, such as the arms and back muscles. Their activation should be triggered only after that of the leg muscles, which perform the most demanding work during exercises on the rowing ergometer. For these reasons, the temperature characteristics of different muscle groups activated during such training may differ, as seen in the obtained results.

However, a detailed analysis of the temperature changes occurring in individual areas of interest was performed, which were assigned topologically to the corresponding muscle parts, symmetrical to the sagittal plane, passing through the long axis of the spine. Obtained temperature changes of the most commonly studied muscles showed the minimum temperature at the beginning of training, primarily in phases 1 and 2. During the subsequent phases, the temperature increase was monitored up to resting temperature. It is important that temperature variations do not exceed 0.5 °C in all post-training phases. Statistical analyses did not show any significant differences in the symmetry of right and left muscle areas corresponding to the muscle location temperature.

Based on the body surface temperature changes, we can conclude that. since the training affects the development of a sportsman’s physiological processes, it should also be connected with the efficiency of thermoregulation mechanisms. It is known that. as a result of systematic training, the lactate threshold increases, which allows exercises of higher intensity without significant signs of fatigue [31,32,33]. It is defined as the intensity of exercise above which the lactate concentration in the blood rises above the resting level. Such processes lead to an increase in lactate concentration and a modification in muscle blood flow, so an increase in tissue oxygen efficiency occurs [31,32,33,34,35,36,37,38,39]. The increase in blood flow leads to better oxygenation of tissues and influences the body’s efficiency parameters. As a result, it may significantly affect thermal comfort and the thermoregulation mechanisms in the sportsman’s body and the body’s surface temperature. Therefore, thermal imaging is increasingly widely used in sports medicine [31,32,33,34,35,36,37,38,39]. It seems that thermal imaging can bring benefits like whole or part of the body temperature map changes or some temperature parameters proposed in the literature which describe effects of bodywork due to training [31,32,33,34,35,36,37,38,39]. Moreover, such an imaging method seems quite interesting and innovative due to the possibility of checking induced adaptation processes, which may become indicators of an athlete’s efficiency and protect him from overtraining or serious injuries [31,32,33]. It seems that, soon, thermal imaging may become helpful for training monitoring by using some temperature parameters as indicators of efficiency level and as overtraining predictor [33,34,35,36,37,38,39].

The most commonly observed reaction to physical activity using a rowing ergometer was a decrease in skin temperature in the initial phase of the exercise, which is consistent with the results of other authors [40,41,42]. The reason for such a change is the reaction of the skin’s blood vessels in contracting in response to exercise. After that, the skin temperature at specific regions of the body may primarily increase when blood flow drifts from internal tissues to the skin surface, for dissipating extra core and muscles located underneath heat production due to an increased metabolic activity [5,43,44,45].

Similarly, in our research we observed another increase in temperature in the subsequent phases of the exercise.

No statistically significant differences were obtained between each phase in all studied symmetrical pairs of muscles. It was also confirmed that there were singular cases where, for one phase during training in whole time-temperature changes, the differences were larger than the rest (ROI 1 I 2 in Phase 3 where *p* = 0.08) or even statistically significant. However, there was only one such case, seen in Figure 4 (ROI 3 and 4 in phases 3 and 4, where *p* = 0.04), where significant differences between temperatures were obtained. This may be connected with a quite small group of volunteers and their individual features.

In summarising, it seems that the courses of temperature changes of selected muscle groups and the obtained amplitudes in selected post-training phases may serve as a non-invasive assessment of the body’s efficiency. Indeed, thanks to these, it is possible to assess the symmetry of the work of individual muscle groups; thus, we can monitor the correctness of their work without risking potential injury and damage to the muscle.

The research’s strength is the adherence to the accepted standards for thermographic measurements introduced by the International Thermological Association [19,46]. Moreover, the group of volunteers in the study was precisely selected. Volunteers had to be adults (male) in the smallest possible age range (24 to 30 years). The high level of training required and the completion of at least three training units per week were the hardest requirements. Additionally, these individuals had to maintain this high level of training for at least two years.

On the other hand, much attention was paid to the initial interview, selecting only people who declared good psychophysical condition and were healthy on the day of the examination. Such requirements were not easy to meet, and only six people qualified. However, this strict selection resulted in the high homogeneity of the studied group.

Therefore, the obtained results constitute only a preliminary database describing the thermal parameters of muscle groups during training on a rowing ergometer. We know that our research included a relatively small group of volunteers (*n* = 6), which is the most significant limitation of the presented results. In the future, we plan to expand the size of the group, as well as to compare the muscle work of athletes and people with a low degree of training (amateurs).

## 5. Conclusions

The study showed that thermal imaging makes it possible to assess the symmetry of the work of individual muscle groups.

Based on the temperature functions obtained from the individual regions of interest with respect to the sagittal plane passing through the long axis of the human being, it has been shown that conventional rowing ergometer training causes almost symmetrical work of the right and left sides of the body.

The temperature changes in most of the studied muscle areas showed minimum temperature reached at the training phase—mostly phases 1 and 2. Next, the temperature increase was observed to reach the primary temperature. The temperature variations during the whole test did not exceed 0.5 °C in all post-training phases. Moreover, statistical analysis did not show any significant differences in the symmetry of right and left muscle areas corresponding to the muscle location temperature.

Thermal imaging may be a completely non-contact, safe and innovative method used to verify the symmetry of work of individual muscle segments. It seems that using an automatic comparison of average temperature or temperature difference may bring clues to protecting athletes from overtraining or serious injuries.

## Figures and Tables

**Table 1:** Features of the research group.

Features	Research Group
Age (years)	27 ± 3
Body weight (kg)	73.82 ± 5.58
Growth (m)	1.78 ± 0.08
BMI (kg/m^2^)	23.25 ± 1.10
Fat tissue (%)	15.24 ± 2.80
Distance (m)	855.00 ± 49.18

**Table 2:** Normalised temperature changes in the group of professionals.

ΔT (°C)								σ (°C)							
	Phase								Phase						
ROI	0	1	2	3	4	5	6	ROI	0	1	2	3	4	5	6
**1**	0	−1.36	−2.15	−0.52	−0.57	−0.50	−0.65	**1**	0.74	0.88	0.80	0.45	0.41	0.36	0.38
**2**	0	−1.30	−1.93	−0.32	−0.40	−0.16	−0.49	**2**	0.85	0.86	0.82	0.66	0.50	0.39	0.48
**3**	0	−0.68	−2.07	0.15	0.00	0.01	−0.30	**3**	0.64	0.68	0.78	0.49	0.51	0.57	0.63
**4**	0	−0.53	−2.18	0.39	0.25	0.13	−0.12	**4**	0.68	0.65	0.86	0.46	0.53	0.63	0.68
**5**	0	−1.08	−0.49	−0.19	−0.12	−0.29	−0.37	**5**	0.98	1.23	0.98	1.02	1.05	1.02	1.01
**6**	0	−1.17	−0.46	−0.18	−0.04	−0.17	−0.37	**6**	0.88	1.14	0.88	0.93	1.05	1.00	0.85
**7**	0	−2.15	−2.93	−0.71	−0.58	−0.47	−0.57	**7**	0.63	0.75	0.92	0.80	0.74	0.73	0.73
**8**	0	−2.23	−3.15	−0.71	−0.60	−0.46	−0.63	**8**	0.66	0.72	0.95	0.78	0.73	0.72	0.70
**9**	0	−1.79	−3.22	−0.65	−0.36	−0.25	−0.35	**9**	0.45	0.58	0.71	0.51	0.46	0.44	0.43
**10**	0	−1.99	−3.27	−0.58	−0.38	−0.24	−0.33	**10**	0.40	0.56	0.62	0.48	0.43	0.39	0.38
**11**	0	−2.12	−3.55	−0.80	−0.54	−0.30	−0.47	**11**	0.47	0.75	0.81	0.65	0.56	0.52	0.57
**12**	0	−1.70	−0.92	−0.30	−0.12	−0.11	−0.13	**12**	0.46	0.54	0.46	0.48	0.47	0.43	0.42
**13**	0	−1.72	−0.95	−0.38	−0.19	−0.19	−0.23	**13**	0.46	0.51	0.46	0.43	0.43	0.38	0.40
**14**	0	−1.19	−0.79	−0.41	−0.35	−0.43	−0.38	**14**	0.66	0.64	0.56	0.61	0.62	0.57	0.62
**15**	0	−1.23	−0.82	−0.40	−0.41	−0.53	−0.58	**15**	0.68	0.71	0.59	0.60	0.59	0.59	0.63
**16**	0	−0.59	0.15	0.40	0.46	0.44	0.25	**16**	0.84	0.95	0.87	0.83	0.82	0.79	0.83
**17**	0	−0.50	0.16	0.45	0.45	0.46	0.28	**17**	0.91	0.99	0.93	0.79	0.80	0.79	0.78
**18**	0	−1.51	−0.72	−0.33	−0.34	−0.48	−0.59	**18**	0.82	0.87	0.86	0.74	0.67	0.67	0.69
**19**	0	−1.59	−0.74	−0.36	−0.40	−0.43	−0.64	**19**	0.75	0.88	0.80	0.75	0.69	0.69	0.69
**20**	0	−1.50	−0.79	−0.31	−0.08	−0.13	−0.19	**20**	0.51	0.49	0.40	0.63	0.42	0.43	0.47
**21**	0	−1.64	−0.84	−0.24	−0.09	−0.13	−0.21	**21**	0.49	0.44	0.45	0.40	0.39	0.37	0.42

## Data Availability

Data sharing is not applicable to this article.
